# The federal spending power: Building forward after the pandemic

**DOI:** 10.1177/08404704211012725

**Published:** 2021-05-17

**Authors:** Fiona A. Miller

**Affiliations:** 1 Centre for Sustainable Health Systems, Institute of Health Policy, Management and Evaluation, 7938 University of Toronto, Toronto, Ontario, Canada.

## Abstract

The phrase, “the federal spending power,” identifies the federal government’s ability to spend in areas beyond its constitutional authority to legislate—a power that has supported the development of a national system of universal healthcare coverage in Canada. Even before the COVID-19 pandemic, this power was critical to the expansion of Canada’s narrow but deep basket of universally covered services. The challenges exposed by the pandemic mean that still more federal investment will be required. Yet for traditionalists, the material basis of this power is now constrained: the federal government may possess the constitutional authority to invest, but it lacks the fiscal capacity; some form of belt tightening—even austerity—will be necessary. As debates over public spending intensify, health leaders will need to address these questions. Depending on how they do so, health leaders will either support or detract from a healthy recovery.

## Introduction

The phrase, “the federal spending power,” is well established in Canadian health policy; it speaks to the federal government’s ability to spend in areas beyond its constitutional authority to legislate.^1^ Although contentious in some quarters, this power has survived challenge in the courts and has played a critical role in the emergence and preservation of a national system of universal healthcare coverage—that is, Canada’s system of Medicare.^2^ Even before the pandemic, the expanded use of this spending power was imperative if long-standing efforts to grow the “narrow but deep” Medicare basket were to stand a chance of success.^3^ With the challenges that the pandemic has exposed, and Canada’s aim to “build back better,”^4^ even more federal investment will be required. Yet for some, the economic consequences of the pandemic call into question the material basis of this spending power. Although the federal government may possess the constitutional authority to invest, it may lack the fiscal capacity. Indeed, from this perspective, some form of belt tightening—even austerity—will be necessary.^5^ The aim of this article is to suggest otherwise.

We first make the case for investment, briefly reviewing several weaknesses that the pandemic has exposed—within and beyond Canada’s health system. As well, we note the accelerating social, economic, and ecological challenges that will continue to test the health system’s resilience in the years and decades ahead. Canada needs to build forward, not just build back. We next review the several reasons why such investment is actually feasible despite assumptions that the cupboard is bare. We begin with traditional economic arguments about the financial sustainability of government debt in Canada and the economic and social risks of austerity. We then turn to the more recent economic arguments that have grown in prominence since the global financial crisis of 2008, and which have been made more visible by decisive monetary policy interventions by the Bank of Canada during the COVID-19 pandemic.

Throughout, we argue for the distinctive importance of the federal spending power. This is not to ignore the central role of the provinces and territories in regulating and delivering health and social services, but to acknowledge the unique fiscal and monetary capacity of the federal government, and the uniquely important role the federal government plays in fostering and enabling shared national projects.

For health leaders, these are issues of more than abstract significance. As debates over public spending intensify, health leaders will play important roles as advocates for investments in healthcare—and for the social and economic investments that support population health and health equity. Indeed, depending on what position they take regarding the scope and significance of the federal spending power, health leaders can either support—or detract from—a healthy recovery.

### The need for investment

In some respects, Canada’s health system has performed well in the face of the COVID-19 pandemic.^6^ Canadians have been able to gain access to COVID-19 care; they have benefited from a rapid transition to virtual care; and major restrictions on access to essential and elective non-COVID-19 health services have been avoided.^7^ At the same time, the system’s over-emphasis on physician and hospital services has led to predictable problems within the provincial and territorial patchwork of extended health services, where Medicare terms and conditions do not apply.^8^

The most flagrant failures have occurred within the systems that should support older adults and those with pre-existing health conditions, through care in the home and community and long-term residential care.^9^ Services to support the frail elderly in their homes and communities have been insufficient.^10^ And the majority of Canada’s COVID-19 deaths have occurred in long-term residential care—the highest proportion among comparator countries.^11^ In addition, Canada’s reliance on employment-based private health insurance to ensure access to outpatient pharmaceuticals has shown obvious limitations. Many people who lost their jobs due to COVID-19 have also lost their benefits.^12^ According to the Heart and Stroke Foundation, “Canadians are twice as likely to have lost prescription drug coverage than to have gained it over the past year.”^13^ Pharmacare—much promised and little delivered—remains as important as ever.^14^

Second, the pandemic has brought attention to the fragility of the globalized medical product supply chain.^15^ This is true everywhere, though with some particularly Canadian weaknesses. Canada struggled to produce and certify personal protective equipment, such as N95 respirators, when global supply was unavailable or insufficient.^16,17^ And though Canada proudly signed multiple advance purchase agreements for vaccines,^4^ the country has been unable to secure a timely supply.^18^ Both weaknesses are the predictable result of a decades-long process of effective “de-industrialization”—in the healthcare marketplace and beyond.^19^ A rejuvenation of industrial policy,^20^ building back some manufacturing, standard-setting and certification infrastructure in critical sectors will be an essential post-pandemic project. In addition to greater resilience, relocalizing some elements of the supply chain promises a wide range of economic, social, and environmental benefits.^19^ The United States is moving decisively to revitalize critical supply infrastructure, including for medical products,^21^ and Canada cannot afford to ignore the opportunities and risks that these global shifts have created. Yet rebuilding the capacity that Canada once possessed^22^ will be expensive and challenging and will necessitate a much more active and inclusive industrial policy than governments in Canada have been disposed to support in recent decades.^23^

Third, the pandemic has exposed deep-seated socio-economic inequalities, which must be addressed in efforts to “build back fairer.”^24,25^ The likelihood of infection from COVID-19, and the risk of poor outcomes, is not equally distributed. It is skewed by the social determinants of health, such as housing and employment, as well as the incidence of prior health conditions, which is itself patterned by social opportunity.^26^ As well, the health consequences of containment measures such as lockdown have been unequally felt, given asymmetric risks of job and income loss, housing adequacy, access to green space, or essential worker status.^26,27^ Even before the pandemic, inequalities in mortality between the highest and lowest income quintiles in Canada had increased in recent decades, as they had in Europe and the United States.^28^ And the economic inequalities that underpin these population health impacts have only worsened during the pandemic.^29^ Since March, Canada’s 44 billionaires have increased their wealth by almost $63.5 billion (CAD).^30^ Economic inequality to this extreme is unjust unto itself. It also weakens social solidarity and is corrosive of democratic institutions.^31^ Meanwhile, the climate crisis, biodiversity loss, soil depletion, and a range of other ecological threats to earth’s biophysical systems pose grave threats to human health and social functioning.^32,33^ As with the pandemic, these threats will harm individuals and communities in unequal ways.^34^ Indeed, equity and ecological sustainability are the “two defining and interdependent challenges of our age.”^35^ This means that the post-COVID era cannot simply aim at building back; the country must build forward in ways that restructure social, economic, and environmental relations.^36^

### The capacity for investment

Even recognizing their compelling nature, calls for increased investment must contend with the fact that government deficits (and accumulated debts) are now at elevated levels. Government investments to support Canadian households and businesses have skyrocketed during the pandemic, even as provincial and federal revenues have declined. The federal government’s projected deficit for 2020 to 2021 is $381.6 billion, increasing the debt to Gross Domestic Product (GDP) ratio from 31.2% in 2019 to 2020 to 52.6% at its expected peak, in 2021 to 2022.^4^ For some commentators, these growing debts mean that “Canada is robbing its kids.”^37^ Nothing could be further from the truth.

Canada’s deficits and debts do not mean that greater investment is not possible. This is, first, for traditional reasons: Government debt in Canada is not unsustainable. Interest rates are likely to remain below nominal growth rates, permitting Canada’s economy to steadily out-grow its debt.^38^ Further, low interest rates have been locked-in for the long term. To an unprecedented extent, the federal government has borrowed money in the form of long-term bonds (eg, for 10 and 30 years). Thus, even as deficits have increased in the past year, borrowing costs have actually declined.^4^ Even without this, Canada’s debt to GDP ratio remains low relative to comparator countries. This is Canada’s “low debt advantage,” which the federal government is eager to maintain.^4^ That said, the budgetary outlook for most provinces is considerably less rosy than that for the feds.^39^ Rising costs, particularly for healthcare, will not necessarily be matched by rising revenues, leaving a growing fiscal gap on current trajectories.^40,41^ Fortunately, the projected federal surplus can more than offset the provincial gap,^41^ even without needed reforms to Canada’s tax system to make it fairer.^42^ Canada is already an international outlier in the limited extent to which the federal government contributes to the expenditures of subnational governments,^43,44^ with federal transfers estimated to cover only 23.5% of provincial healthcare expenditures.^43^ Correcting this, by increasing federal fiscal transfers to the provinces and territories (as has been demanded by Canada’s premiers^45^), will be an essential element of sustainable government budgets going forward.

In addition to these classic calculations, there is growing recognition of the harms of austerity,^46^ which risks economic recovery, socio-economic opportunity, and population health—as demonstrated by the harsh austerity measures imposed in Europe after the global financial crisis.^47^ Even the bastions of neo-liberal capitalism—the IMF and World Bank—have recommended that governments continue to use debt-financing to invest in their economies and not move too quickly to slow the pace of these investments.^48^ On these measures, Canada is already missing the mark, having been identified by *The Economist* as “the country most exposed to austerity.”^49^ Indeed, the federal government’s intent to sustain its “low debt advantage” looks increasingly unwise. With low inflation, a floating currency, and modest amounts of foreign debt, the federal government is moving too quickly to reduce its deficits. Moreover, since debt sustainability on these terms is about expenditure relative to revenue, the real question is not how much Canada is spending, but what that spending is buying—and specifically, whether the federal spending power is positioning the country for success by investing aggressively in new social, environmental, and industrial strategies.^50^ Arguably, here, the answer is no.^51^

An added twist to these traditional arguments is the shifting nature of monetary policy, given the increased willingness of central banks “to serve as lenders of first resort to governments.”^50^ When the pandemic struck, the Bank of Canada moved quickly to reduce its policy interest rate—which determines the cost of short-term borrowing—to 0.25%. Then, to further reduce borrowing costs and in service of a target inflation rate of 2%, the Bank initiated a massive round of large-scale asset purchases. Such “quantitative easing” (QE) involves purchases by the Bank in the secondary market of bonds that were previously issued by the Government of Canada.^52^ Indeed, starting in April 2020, the Government of Canada Bond Purchase Program spent a minimum of $5 billion weekly buying Government of Canada bonds. Then, to put more direct downward pressure on long-term interest rates, the Bank shifted its purchases to longer term, 5-year bonds, and has promised additional measures (eg, yield-curve control), should these efforts prove insufficient.^53^ In addition to classic QE—buying back previously issued bonds on the secondary market from commercial banks and other investors—the Bank of Canada has increased its routine purchase of newly issued bonds.^54^ Indeed, as a matter of policy, the Bank of Canada purchases at least 20% of all Government of Canada bonds at the time of their issue, on a non-competitive basis.^55^ Taken together, the Bank of Canada—a crown corporation wholly owned by the federal government—is expected to hold 60% of the Canadian bond market by the end of 2021.^50^ This has raised some eyebrows. Perhaps, “the Bank’s monetization policy is innocuous”?^56^ Or perhaps, Canada is a worrying “lab experiment for Modern Monetary Theory”?^57^

Modern money is “fiat” money. Its value is not secured by being convertible into a commodity such as gold; it is secured by government authority. Modern monetary theory attempts to explain the monetary sovereignty this confers for currency-issuing governments such as Canada’s federal government and prescribes a set of policy measures to democratize and fairly distribute the benefits of this power.^58^ This theory does not suggest that there are *no* limits to the spending power of monetary sovereigns. Rather, it argues that attention to limits should shift *away* from money, which is a human creation, to the real resource constraints that threaten societies and economies—limits to human, material, and ecological resources.^59^ In this respect, modern monetary theory is one of several contributions to debates about economic growth since the global financial crisis in 2008. In the face of a halting post-crisis recovery and accelerating and unsustainable degrees of inequality and environmental degradation, contemporary thinking aims to put human well-being and planetary health at the core,^46^ and to challenge “growth,” particularly as measured by GDP, as an adequate indicator of economic performance and social progress.^60^ Such ideas add new meaning to the phrase, “the federal spending power,” offering a rationale for the greater policy room possessed by the federal government (the currency issuer) relative to provincial and territorial governments (currency users). These ideas also point to the need for a wider dashboard of indicators in assessments of what any spending should aim to accomplish^61^ and highlight the risk of under-investment: that a failure to invest is, quite simply, unsustainable.

## Conclusion

The federal spending power has a long and storied policy history in Canada. In the context of a deeply decentralized federation, with constitutional authority divided between both levels of government, a nationally consistent, generous, and universally accessible model of health insurance was only made possible through “shared-cost federalism.”^2,62^ Yet the system built through shared costs has been buffeted by shifts in those pooled investments, which have left the provinces with responsibility for a greater proportion of national expenditure on healthcare than the federal government,^63^ representing an increasing share of provincial/territorial government program expenditures (37% of the total on average).^64^ Even before the pandemic, Canada’s provinces and territories were not well placed to carry those extra expenditures.^41^ Their fiscal challenges have been amplified by the economic and social dislocations of the pandemic and the problems it has exposed within and beyond Canada’s health system.^44^

New investment is needed to expand Canada’s narrow but deep system of universal healthcare and to invest in building the country forward, for more sustainable social, economic, and environmental ways of operating.^65^ Both traditional and more contemporary arguments can be marshalled to suggest that the federal government (and not the provinces) has the spending power to pursue an ambitious, strategic investment agenda. But sufficient federal investment is far from assured.^5^ Healthcare’s prominence in provincial budgets means that health leaders are likely to play an important role in debates about federal investment.^66^ If they are not careful, however, Canada may secure investment in healthcare instead of—rather than in addition to—investment in the social, environmental, and economic conditions that support health. This is not a trade-off worth making. More importantly, given the scope of the federal spending power, this is not a trade-off that needs to be made.
